# A robust 11-genes prognostic model can predict overall survival in bladder cancer patients based on five cohorts

**DOI:** 10.1186/s12935-020-01491-6

**Published:** 2020-08-20

**Authors:** Jiaxing Lin, Jieping Yang, Xiao Xu, Yutao Wang, Meng Yu, Yuyan Zhu

**Affiliations:** 1 Department of Urology, The First Hospital of China Medical University, Shenyang, 110001 Liaoning China; 2 Department of Pediatric Intensive Care Unit, The Shengjing Hospital of China Medical University, Shenyang, 110001 Liaoning China; 3 Department of Reproductive Biology and Transgenic Animal, China Medical University, Shenyang, 110001 Liaoning China

**Keywords:** Bladder cancer, Cox regression, Prognostic model, Overall survival

## Abstract

**Background:**

Bladder cancer is the tenth most common cancer globally, but existing biomarkers and prognostic models are limited.

**Method:**

In this study, we used four bladder cancer cohorts from The Cancer Genome Atlas and Gene Expression Omnibus databases to perform univariate Cox regression analysis to identify common prognostic genes. We used the least absolute shrinkage and selection operator regression to construct a prognostic Cox model. Kaplan–Meier analysis, receiver operating characteristic curve, and univariate/multivariate Cox analysis were used to evaluate the prognostic model. Finally, a co-expression network, CIBERSORT, and ESTIMATE algorithm were used to explore the mechanism related to the model.

**Results:**

A total of 11 genes were identified from the four cohorts to construct the prognostic model, including eight risk genes (SERPINE2, PRR11, DSEL, DNM1, COMP, ELOVL4, RTKN, and MAPK12) and three protective genes (FABP6, C16orf74, and TNK1). The 11-genes model could stratify the risk of patients in all five cohorts, and the prognosis was worse in the group with a high-risk score. The area under the curve values of the five cohorts in the first year are all greater than 0.65. Furthermore, this model’s predictive ability is stronger than that of age, gender, grade, and T stage. Through the weighted co-expression network analysis, the gene module related to the model was found, and the key genes in this module were mainly enriched in the tumor microenvironment. B cell memory showed low infiltration in high-risk patients. Furthermore, in the case of low B cell memory infiltration and high-risk score, the prognosis of the patients was the worst.

**Conclusion:**

The proposed 11-genes model is a promising biomarker for estimating overall survival in bladder cancer. This model can be used to stratify the risk of bladder cancer patients, which is beneficial to the realization of individualized treatment.

## Background

Bladder cancer is the tenth most common cancer in the world. It is more common in men than in women, and the morbidity and mortality rate in men is four times higher than that in women [1]. A significant risk factor for bladder cancer is smoking, with half of all cases are linked to smoking [2, 3]. About 75% of patients with non-muscular invasive bladder cancer are treated by radical tumor resection, followed by intravesical instillation of Bacille Calmette-Guérin vaccine. Approximately 25% of patients have muscular invasive or metastatic bladder cancer, and are treated with radical cystectomy and neoadjuvant chemotherapy [4]. Bladder cancer is a complex disease. Although many clinical factors and molecular markers have been identified that can predict prognosis [5], these have low accuracy, and it does not have universal applicability.

With the continued development of gene sequencing technology and expansion of public databases, it is possible to take advantage of biological information to mine sequencing data and identify biomarkers. This method can utilize large sample sizes with less investment, making it an important new direction to screen disease biomarkers. Of available databases, the Cancer Genome Atlas (TCGA, https://cancergenome.nih.gov/) database is an authoritative oncology database, and the Gene Expression Omnibus (GEO, http://www.ncbi.nlm.nih.gov/geo/) database stores curated gene expression datasets. Many studies have constructed a multi-queue verification model based on these two databases, such as non-small cell lung cancer [6, 7], and ovarian cancer [8]. Prognostic models provide effective guidance for doctors and patients to make optimal treatment decisions. However, in the study of the bladder cancer model, many models can only be verified in two or three cohorts [9, 10] and do not have clinical extensibility.

In this study, gene expression and clinical data related to bladder cancer were obtained from TCGA and GEO databases, and common prognostic genes were screened by univariate Cox proportional hazard regression. This prognostic model of bladder cancer was constructed by least absolute shrinkage and selection operator (Lasso) regression and then verified using five cohorts. This robust model can help patients with bladder cancer to achieve individualized treatment.

## Materials and methods

### Data obtaining and processing

To reduce the error of the data, we searched the TCGA and GEO databases for bladder cancer cohorts with a sample size of more than 100, and these cohorts need to include survival status and survival time. We found a total of five cohorts. The raw RNA sequencing and clinical data of bladder urothelial carcinoma (BLCA, n = 412) were obtained from TCGA database, and the raw RNA sequencing and clinical data of GSE13507 (n = 165) [11], GSE32548 (n = 146) [12], GSE32894 (n = 308) [13] and GSE48075 (n = 142) [14] from the GEO database. These five cohorts were analyzed on the Illumina sequencing platform. In R Programming Language software, the R package “edgeR” [15] was used to standardize the raw RNA expression matrix and obtain the corresponding log values.

### Construction of prognostic model

The Cox proportional hazard regression model was applied to perform univariate Cox proportional hazard analysis of all genes in TCGA-BLCA, GSE13507, GSE32548, and GSE32894 cohorts. The hazard ratio (HR) from univariate Cox regression analysis was used to select the genes that were positively or negatively related to prognosis. A gene with HR > 1 was considered a risk gene, and a gene with HR < 1 was considered a protective gene; statistical significance was defined as p < 0.05. The genes with HR > 1 and p < 0.05 were selected from the four cohorts, and then risk genes were obtained by overlapping four groups of genes. Similarly, genes with HR < 1 and p < 0.05 were selected for the four cohorts and combined to obtain the set of protective genes. A Venn diagram was constructed using the online tool Bioinformatics and Evolutionary Genomics (http://bioinformatics.psb.ugent.be/webtools/Venn/). The identified risk and protective genes make up a set of prognostic genes.

The data from TCGA-BLCA as a training set was used to construct a prognostic model. To simplify the model, the genes were selected by univariate Cox regression analysis with a *p* value less than 0.01. The R package “glmnet” [16] and “survival” were used to do Lasso regression to further screen genes and construct a Cox module. First, the function “glmnet” was randomly simulated 1000 times to construct the model and establish the relationship between lambda (punishment coefficient) and regression coefficients (coef). A higher value of lambda corresponds to greater punishment. With the increase of lambda, some gene coef become zero, indicating that the expression of the gene will not affect the model, so this gene can be removed from the model. Then the function “cv.glmnet” was randomly simulated 1000 times for cross-validation (CV). CV is usually divided into hold-out, k-fold and leave-one-out CV. The function used k-fold CV, and k took the default parameter 10. In tenfold cross validation, the data set is divided into 10 equal parts, and then nine part are tested as training sets and one is used as the validation set. The deviance of the 10 tests were used to estimate the accuracy of the model. When the deviance is minimum, the model is the best, and the coef of the model can then be obtained by using the corresponding lambda value. Finally, we obtained the genes and the corresponding coef to build the model. The prognostic model was defined as: Risk score = ∑n_i_ (exp_i_ · coef_i_) (where n is the number of genes, exp_i_ is the expression of the ith gene, and coef_i_ is the regression coefficient of the ith gene). The algorithm can prevent over-fitting of the model, remove highly co-expressed genes, and finally construct a simplified model. Using the obtained model, we calculated the risk score of each patient in the four cohorts.

### Kaplan–Meier analysis

R packages “survival” and “survminer” were used for Kaplan–Meier analysis, and the function “res.cat” was used to find the best cut-off value of factors. The cut-off was used to divide the sample into a high-risk group and a low-risk group to construct the Kaplan–Meier curve with the smallest p value. The risk score distribution, gene expression, and patient survival status data were plotted using the R package “pheatmap”.

### Receiver operating characteristic curve

Receiver operating characteristic (ROC) curves of 1/3/5 years were plotted and the area under the curve (AUC) values were calculated using the R package “survivalROC”.

### Univariate and multivariate Cox regression analysis

The risk scores and clinicopathological factors were analyzed by univariate and multivariate Cox regression analysis using the R package “survival”. The multivariate Cox analysis included age, sex, primary tumor range (T stage), grade, and risk score (TCGA-BLCA and GSE13507 also include stage, lymph node and metastasis).

### Exploration of gene methylation

“CBioPortal for Cancer Genomics” is an open-access open-source resource (https://www.cbioportal.org) for interactive exploration of multiple cancer genomics data sets [17, 18]. Use this tool to query the relationship between gene expression and DNA methylation in the “Bladder Cancer (TCGA, Cell 2017)” dataset. The tool can also download gene methylation data, which can be combined with clinical data for Kaplan–Meier analysis.

### Weighted co-expression network analysis

The set of mRNA genes in the TCGA-BLCA cohort with univariate Cox analysis values less than 0.05 were selected to construct a bladder cancer co-expression network by weighted gene co-expression network analysis (WGCNA). The R package “WGCNA” was used to construct the co-expression network [19]. This method takes advantage of similarities of gene expression and groups the genes with similar expression patterns into the same module, with the idea that genes in the same module may share physiological function. We then explored the relationship between the clinical-factor/risk-score and module, and applied Pearson correlation to determine the module that was most related to the risk score. The key genes were selected by the calculated correlation between genes (module-membership > 0. 8), and the correlation between genes and clinical traits (Gene-significance > 0. 5).

### Pathway and process enrichment analysis

Identified genes were entered into the Metascape database (http://metascape.org) [20] for pathway and process enrichment analysis. The enrichment analysis included “KEGG Pathway, GO Biological Processes, Reactome Gene Sets, Canonical Pathways, and CORUM” to evaluate the potential biological functions and pathways of the selected genes.

### CIBERSORT and ESTIMATE algorithm

CIBERSORT (Cell-type Identification By Estimating Relative Subsets Of RNA Transcripts) is a bioinformatics algorithm to calculate cell composition from gene expression profiles of complex tissues [21]. The combination of CIBERSORT and LM22 (leukocyte signature matrix) can be used to calculate the content of 22 kinds of human leukocyte subsets. We used the R package “CIBERSORT” to calculate the number of immune cells in each sample of the TCGA-BLCA cohort. ESTIMATE (Estimation of STtromal and Immune cells in MAlignant Tumours using Expression data) is a tool that uses gene expression trends to infer the fraction of stromal and immune cells in tumor samples [22]. The immune score of each patient in TCGA-BLCA was calculated by the R package “estimate”. Immune score represents the content of immune cells, and the higher the score, the higher the cell content.

### Statistical analysis

All the statistical analyses were carried out by using R Programming Language software (Rx64 3.5.1). All R packages were obtained from CRAN (https://cran.r-project.org) or BioConductor (http://www.bioconductor.org). The two groups were compared by the Wilcoxon test, and comparison between multiple groups was performed by Kruskal–Wallis test. Statistical significance was defined as p < 0.05. Difference scatter plots were constructed using the R package “beeswarm”. We used the R package “vioplot” to draw violin pictures and the R package “corrplot” to draw correlation heat map.

## Results

### Data processing and research process

**Fig. 1:**
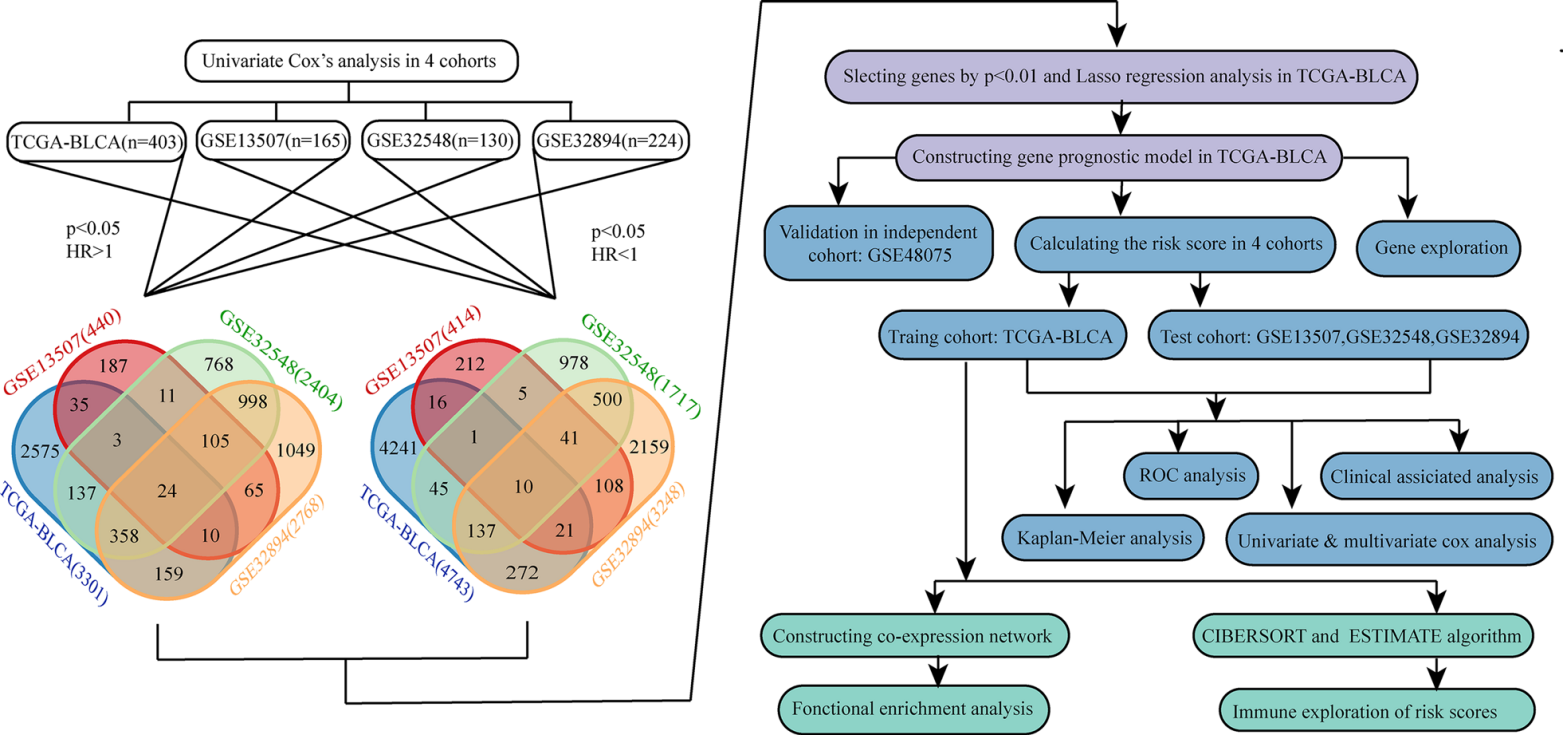
Flow chart of analysis

We obtained the raw RNA sequencing and clinical data of TCGA-BLCA (n = 412), GSE13507 (n = 165), GSE32548 (n = 146) and GSE32894 (n = 308). We utilized data only from patients associated with RNA sequencing data, survival time, survival status, and primary tumor for further analysis. The basic clinical information of the remaining patients is summarized in Table 1, the sample sizes of these four cohorts are all greater than 100. The grade of bladder cancer is closely related to recurrence and invasive behavior. Two grading methods were used in these four cohorts. TCGA-BLCA and GSE13507 used the WHO grading standard of 2004, which was divided into PUNLMP (Papillary urothelial neoplasms of low malignant potential), low grade, and high grade. GSE32548 and GSE32894 used the WHO grading standard of 1999, which was divided into grade 1 (G1), grade 2 (G2), and grade 3 (G3). The research process is shown in Fig. 1.

**Table 1:** Basic clinical information for the four cohorts

Clinical factors	TCGA_BLCA		GSE13507		GSE32548		GSE32894	
	n = 403	%	n = 165	%	n = 130	%	n = 224	%
Age								
≦ 60	107	26.55	46	27.88	27	20.77	46	20.54
> 60	296	73.45	119	72.12	103	79.23	178	79.46
Gender								
Male	298	73.95	135	81.82	99	76.15	163	72.77
Female	105	26.05	30	18.18	31	23.85	61	27.23
T stage								
< T2	4	0.99	104	63.03	91	70	173	77.23
≧ T2	366	94.29	61	36.97	38	29.23	51	22.77
Grade (WHO2004)								
Low	20	4.96	105	63.64	–	–	–	–
High	380	94.29	60	36.36	–	–	–	–
Grade (WHO1999)								
G1	–	–	–	–	15	11.54	45	20.09
G2	–	–	–	–	40	30.77	84	37.50
G3	–	–	–	–	75	57.69	93	41.52
Vital status								
Alive	248	61.54	96	58.18	105	80.77	199	88.84
Dead	155	38.46	69	41.82	25	19.23	25	11.16
Follow-up (mean ± SD, year)								
	2.10 ± 2.23		3.98 ± 3.10		4.14 ± 2.38		3.28 ± 2.10	

*SD* standard deviation

### Construction of prognostic model

**Fig. 2:**
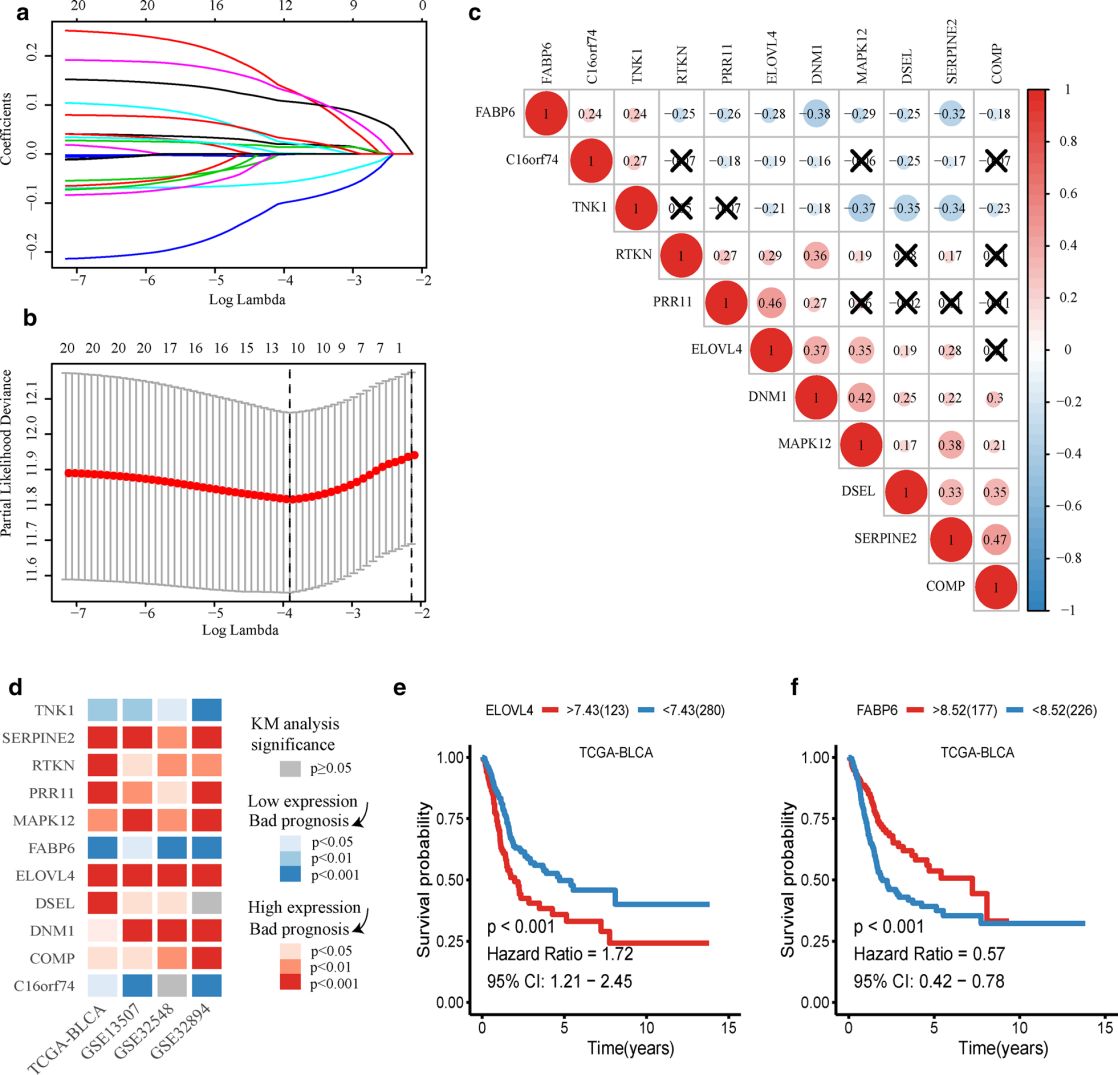
Lasso Cox and Kaplan–Meier analysis. **a** Lines of different colors represent different genes. With the increase of lambda value, the coef of some genes become zero, indicating that they do not affect the model. **b** The deviance of the cross validation. When the partial likelihood deviance is minimum, the corresponding model is the best. **c** The co-expression heat map of 11 genes. Red indicates a positive correlation, blue indicates a negative correlation, and the cross indicates no statistical significance. **d** The heatmap of Kaplan–Meier analysis results of 11 genes. Red means high expression of the gene lead to worse prognosis, blue means low expression of the gene lead to worse prognosis, grey means there are no significance of the analysis, p < 0.05 means it is statistically significant. The darker of the color shows the smaller of the p-value. **e** Kaplan–Meier analysis of ELOVL4 in TCGA-BLCA. **f** Kaplan–Meier analysis of FABP6 in TCGA-BLCA

Univariate Cox proportional hazard analysis was carried out in TCGA-BLCA, GSE13507, GSE32548, and GSE32894 cohorts. There were 3301 genes in TCGA-BLCA, 440 genes in GSE13507, 2404 genes in GSE32548, and 2768 genes in GSE32894 that met the criteria (HR > 1 and p < 0.05). There were 4743 genes in TCGA-BLCA, 414 genes in GSE13507, 1717 genes in GSE32548, and 3248 genes in GSE32894 that met the criteria (HR < 1 and p < 0.05). Combining the four datasets allowed identification of 24 risk genes and 10 protective genes (Fig. 1). Because of the large sample size of TCGA-BLCA, we used this cohort to build the prognostic model. First, 24 genes with univariate Cox p-values less than 0.01 in TCGA-BLCA were selected. Then the 24 genes were analyzed by Lasso regression analysis (Fig. 2a), when the number of genes in the model was 11, the deviance was the smallest (Fig. 2b). According to the lambda value, the corresponding coef of the selected 11 genes could be determined. The prognostic model could then be constructed by using the corresponding coef of the 11 genes. To see more intuitively whether these genes are collinear, we analyze the co-expression of these genes. As shown in the Fig. 2c, the co-expression index of none of these two genes is greater than 0.5. Finally, we successfully constructed a prognostic module: Risk score = SERPINE2 * 0.02 + PRR11 * 0.13 + FABP6 * (− 0.000318) + C16orf74 * (− 0.0564) + DSEL * 0.107 + DNM1 * 0.0142 + COMP * 0.0223 + TNK1 * (− 0.0972) + ELOVL4 * 0.00152 + RTKN * 0.126 + MAPK12 * 0.0304. The basic information and coef values of the 11 genes are listed in (Additional file 1: Table S1). The average expression values (Transcripts per million) of genes in the four cohorts are greater than 1, which is of practical significance for detection (Additional file 2: Table S2). The results of univariate regression analysis of these 11 genes in 4 cohorts are shown in Additional file 3: Table S3.

### Kaplan–Meier analysis of 11 genes

Eleven genes were taken Kaplan–Meier analysis in 4 cohorts. Using the heat map to show the results of the study (Fig. 2d), except for DSEL in GSE32894 and C16orf74 in GSE32548, the other analyses were statistically significant (p < 0.05). SERPINE2, RTKN, PRR11, MAPK12, ELOVL4, DSEL, DNM1, and COMP showed that the prognosis of patients with high expression was worse, and the analysis of ELOVL4 in TCGA-BLCA was taken as an example (p < 0.001, Fig. 2e). TNK1, FABP6, and C16orf74 showed that the prognosis of the low expression group was worse, and the analysis of FABP6 in TCGA-BLCA was taken as an example (p < 0.001, Fig. 2f).

### The degree of DNA methylation of TNK1 and C16orf74 was negatively correlated with gene expression

DNA methylation can regulate gene expression. We explored the relationship between expression and methylation of these 11 genes (Additional file 4: Figure S1a–k). The results showed that there was a negative correlation between TNK1 gene methylation and gene expression (Spearmen cor = − 0.51, p = 1.44e−28), so did as C16orf74 (Spearmen cor = − 0.52, p = 8.97e−29). Then, we took TNK1 and C16orf74 methylation data combined with clinical data for Kaplan–Meier analysis. We found that the degree of methylation of these two genes can predict the prognosis of bladder cancer (p < 0.05, Additional file 4: Figure S1i, m), and the prognosis is worse in the case of hypermethylation. The expression of TNK1 and C16orf74 is inhibited by hypermethylation, which leads to a worse prognosis of bladder cancer.

### Verification of the prognostic model

**Fig. 3:**
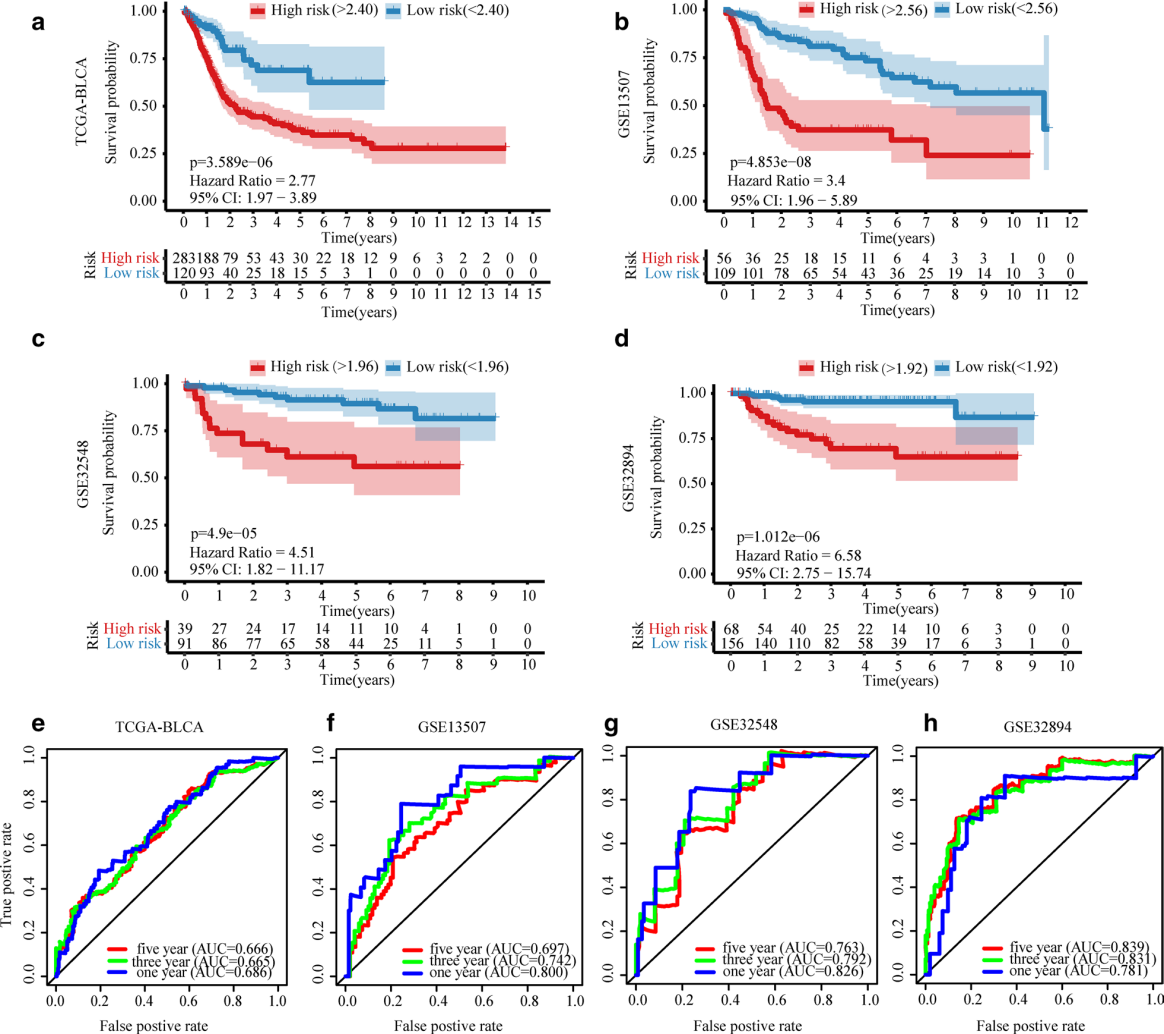
The Kaplan–Meier analysis and ROC curves of the risk score. Kaplan–Meier curves of TCGA-BLCA (**a**), GSE13507 (**b**), GSE32548 (**c**), and GSE32894 (**d**). Red indicates high-risk group and blue indicates low-risk group. p < 0.05 means it is statistically significant. CI: confidence interval. ROC curves of TCGA-BLCA (**e**), GSE13507 (**f**), GSE32548 (**g**), and GSE32894 (**h**) in 1/3/5 years, and their corresponding AUC values

The prognostic model was used to calculate the risk scores of each patient in the training set (TCGA-BLCA) and three test sets (GSE13507, GSE32548, and GSE32894). We identified the best cut-off value with a risk score of 2.40 for TCGA-BLCA. Using this method, the cut-off values of GSE13507/GSE32548/GSE32894 were 2.56/1.96/1.92. The Kaplan–Meier curves showed that the prognosis of patients with high-risk was significantly worse than that of patients with low-risk in the four cohorts (p < 0.001, Fig. 3a–d). The Receiver Operating Characteristic (ROC) curves of the four cohorts were drawn: the 1/3/5 year Area Under the Curve (AUC) values for the TCGA-BLCA cohort were 0.686, 0.665, and 0.666, respectively (Fig. 3e); those for the GSE13507 cohort were 0.800, 0.742, and 0.697, respectively (Fig. 3f); those for the GSE32548 cohort were 0.826, 0.792, and 0.763, respectively (Fig. 3g); and those for the GSE32894 group were 0.781, 0.831 and 0.839 (Fig. 3h). Additional file 5: Figure S2 shows the risk score distribution, gene expression values, and survival status of patients in both the high-risk group and the low-risk group.

The clinical factors and risk scores of the four cohorts were analyzed by univariate Cox and multivariate Cox regression analysis (Table 2). The results of univariate analysis showed that T stage was more effective in predicting prognosis among the clinical factors, and three cohorts had statistical significance. The risk scores were statistically significant in all four cohorts, and the p value of three cohorts was lower than that of the T stage. In multivariate Cox analysis, risk scores were statistically significant in three cohorts, indicating that the three cohorts were independent of other clinical factors in predicting prognosis. In this analysis, only two cohorts of T stage had statistical significance, so it is obvious that T stage is not as strong as risk score to predict the prognosis. Finally, we compared the risk scores for different grades and T stage in the four cohorts, and found that the risk scores increased with the increase of grade and T stage (p < 0.001, Additional file 6: Figure S3a, b). In the GSE32548 cohort, we compared the risk scores of FGFR3, and TP53 (or with the MDM2 alteration) for wild type and mutant type. Additional file 6: Figure S3c shows that a lower risk score of mutant type than that of wild type for the FGFR3 groups (p < 0.001). In TP53 (or with the MDM2 alteration), the score of mutant type was higher than that of wild type (p < 0.001, Additional file 6: Figure S3c).

**Table 2:** Univariate and multivariate Cox regression analysis of clinical-factors/risk-score with overall survival rate in patients

Variables	Univariate analysis		Multivariate analysis	
	HR (95% CI)	p	HR (95% CI)	p
TCGA-BLCA				
Age	1.04 (1.02–1.06)	*1.20E*−*05*	1.02 (0.99–1.05)	2.69E−01
Gender	1.11 (0.78-1.58)	5.56E−01	1.44 (0.81–2.54)	2.14E−01
Grade	9,608,547.45 (0-Inf)	9.91E−01	5,878,954.98 (0-Inf)	9.96E−01
Stage	1.81 (1.47-2.23)	*2.62E*−*08*	0.89 (0.42–1.88)	7.63E−01
T stage	1.75 (1.37-2.24)	*6.57E*−*06*	1.38 (0.82–2.34)	2.23E−01
Node	1.61 (1.35-1.91)	*8.06E*−*08*	1.44(0.86-2.41)	1.64E−01
Metastasis	3.06 (1.39-6.73)	*5.44E*−*03*	0.88(0.28-2.72)	8.24E−01
Risk score	3.75 (2.43-5.79)	*2.46E*−*09*	4.1(1.89-8.91)	*3.67E*−*04*
GSE13507				
Age	1.07 (1.04–1.1)	*4.53E*−*08*	1.07 (1.04–1.1)	*8.71E*−*06*
Gender	1.56 (0.88–2.77)	1.29E−01	1.57 (0.81–3.03)	1.81E−01
Grade	1.9 (1.49–2.42)	*2.46E*−*07*	1 (0.54–1.86)	9.97E−01
Stage	2.74 (1.69–4.43)	*4.00E*−*05*	1.06 (0.52–2.14)	8.70E−01
T stage	2.05 (1.64–2.58)	*5.06E*−*10*	1.51 (0.82–2.79)	1.83E−01
Node	3.32 (2.23–4.94)	*3.88E*−*09*	2.21 (1.09–4.46)	*2.71E*−*02*
Metastasis	9.9 (4.38–22.37)	*3.64E*−*08*	3.61 (1.2–10.86)	*2.21E*−*02*
Risk score	11.16 (4.22–29.47)	*1.14E*−*06*	1.33 (0.26–6.87)	7.37E−01
GSE32548				
Age	1.04 (0.99–1.08)	9.34E−02	1.06 (1.01–1.1)	*2.00E*−*02*
Gender	0.78 (0.29–2.07)	6.13E−01	0.66 (0.23–1.85)	4.27E−01
Grade	2.26 (1.07–4.77)	*3.26E*−*02*	0.46 (0.15–1.44)	1.83E−01
T stage	3.53 (1.89–6.6)	*7.42E*−*05*	3.97 (1.58–9.99)	*3.41E*−*03*
Risk score	43.5 (6.85–276.49)	*6.37E*−*05*	12.81 (1.06–154.42)	*4.46E*−*02*
GSE32894				
Age	0.98 (0.95–1.01)	1.79E−01	0.97 (0.92–1.01)	1.63E−01
Gender	1.47 (0.55–3.93)	4.45E−01	1.35 (0.49–3.74)	5.61E−01
Grade	7.59 (2.45–23.52)	*4.45E*−*04*	3.58 (1.05–12.13)	*4.08E*−*02*
T stage	0.98 (0.62–1.56)	9.42E−01	1 (0.58–1.7)	9.86E−01
Risk score	140.69 (25.35–780.79)	*1.54E*−*08*	31.03 (3.79–253.97)	*1.36E*−*03*

Italic font means statistically significant

*HR* hazard ratio, *CI* confidence interval, *Inf* infinity

### Seven genes and model were successfully verified in GSE48705

**Fig. 4:**
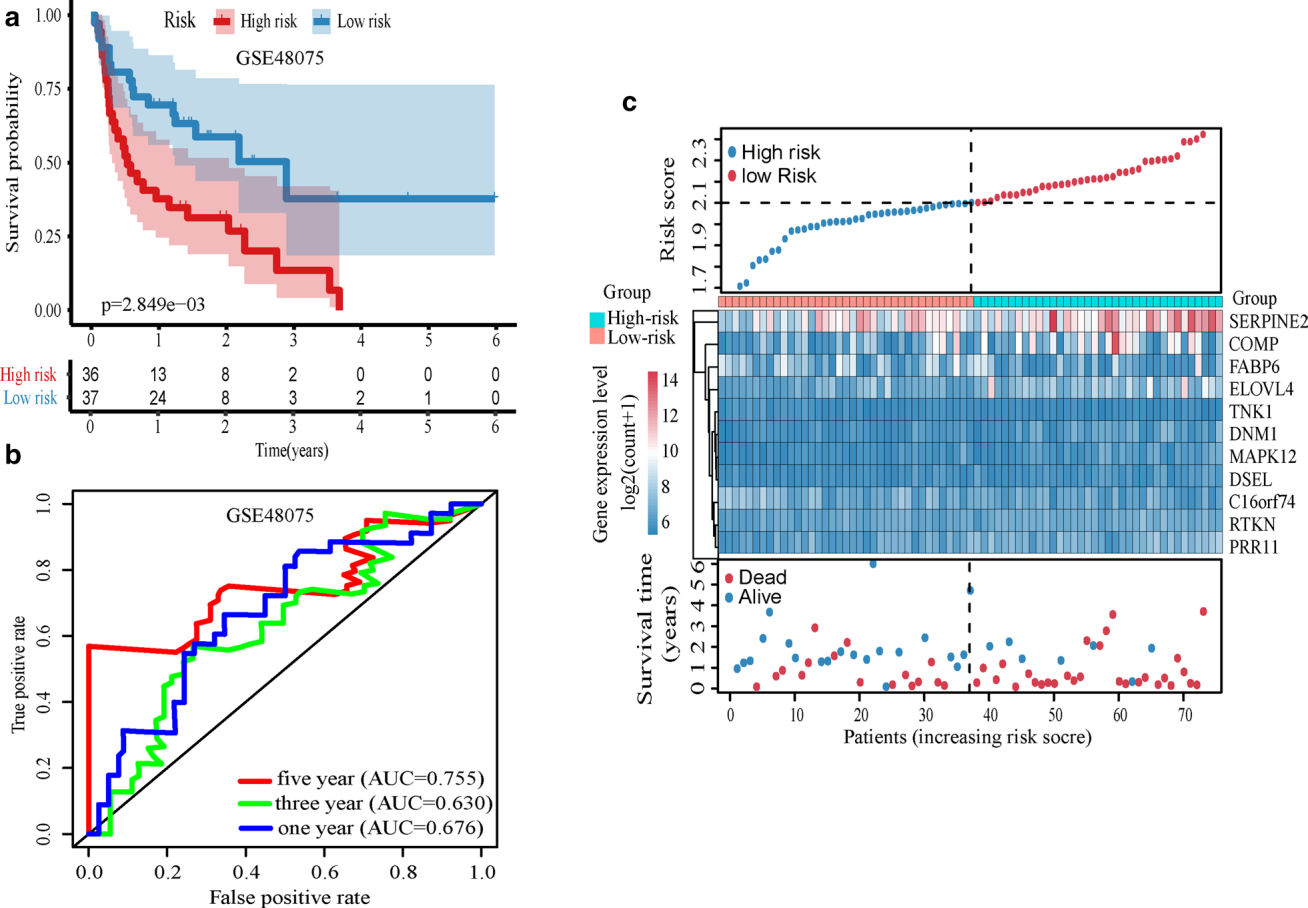
Validation of the model in GSE48075. **a** Kaplan–Meier analysis of the risk-score. **b** ROC curves of risk-score in 1/3/5 years. **c** The risk score analysis, from top to bottom: patient’s risk distribution, gene expression profile and survival status map

We evaluated the prognostic ability of 11 genes and models in GSE48075 (n = 73). The results showed that SERPINE2, RTKN, PRR11, MAPK12, ELOVL4, DSEL, and COMP were statistically significant (p < 0.05, Additional file 7: Figure S4), and the prognosis was worse in the high expression group which was consistent with the analysis result of the previous four cohorts. The risk score of patients was calculated according to the model, and the risk score was analyzed by Kaplan–Meier analysis. The prognosis of the high risk-score group was worse, and the difference was statistically significant (p = 0.0028, Fig. 4a). We drew the ROC curves of the risk score, and the AUC value of 1/3/5 year was 0.676/0.630/0.755 (Fig. 4b). Figure 4c showed the risk score distribution, gene expression values, and survival status of patients between high and low-risk groups.

### Weighted co-expression network analysis and enrichment analysis

**Fig. 5:**
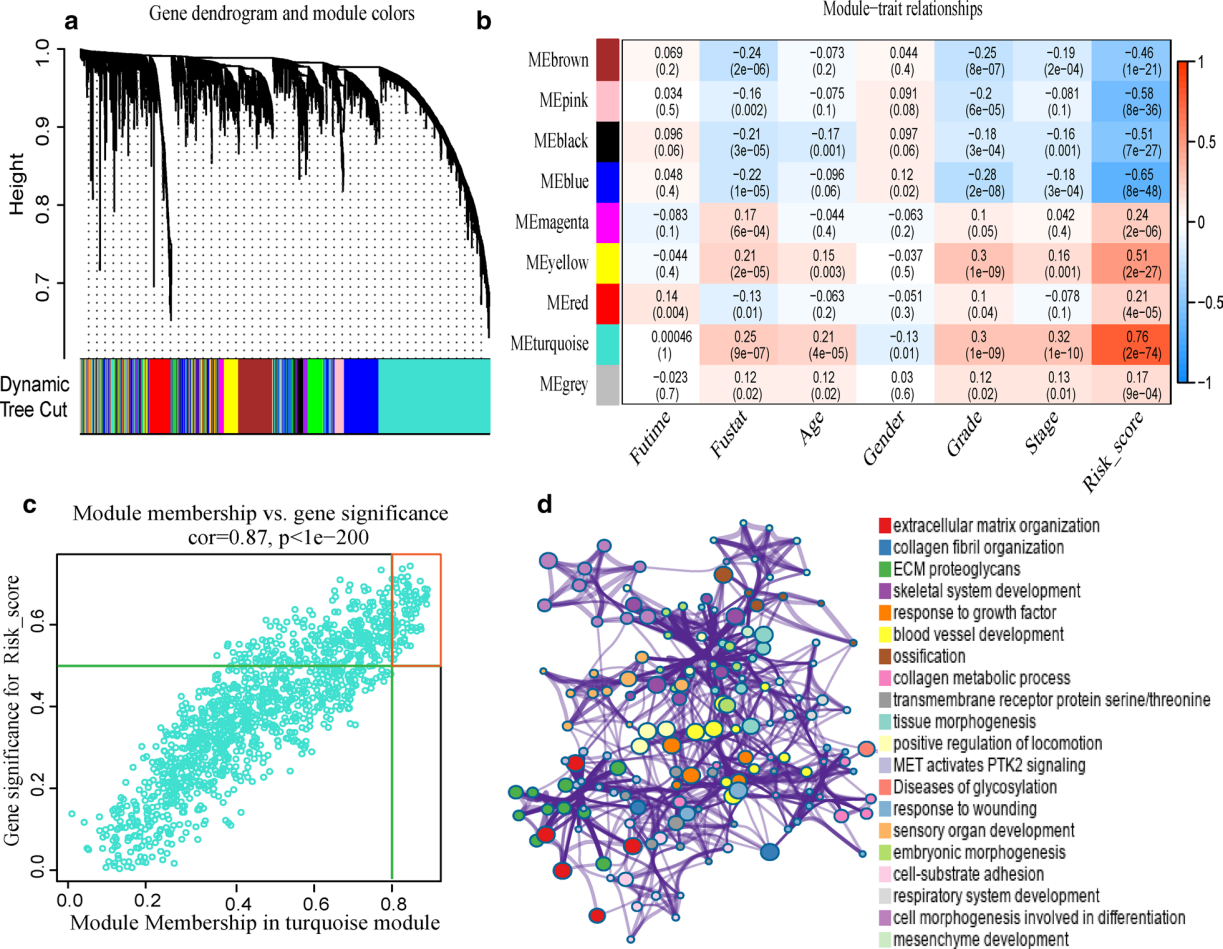
Weighted co-expression network and enrichment analysis. **a** Genes were divided into different modules according to the dynamic cutting tree method, and different colors represent different modules. **b** The heatmap of the correlation between the gene module and clinical traits, p < 0.05 indicates statistical significance. **c** Gene significance and module membership scatter diagrams of the turquoise module. The dots in the red box are represented as key genes. **d** The left side of the picture shows the interaction network map of enriched proteins, and the same color indicates the same enrichment. The right side of picture shows the enriched terms decreasing from top to bottom by the significance of enrichment

The co-expression network was constructed with 3844 coding genes and 403 samples in TCGA-BLCA cohort. First, the expression matrix was transformed into a topological overlap matrix according to β = 4. Then, the genes were divided into different modules (Fig. 5a) using the dynamic pruning tree method. Next, the association analysis of clinical traits and modules (Fig. 5b) showed a high correlation between the turquoise module and risk score (cor = 0.76, p = 2E−74). There was also a high correlation between the turquoise module and survival status (cor = 0.25, p = 9E−07)/grade (cor = 0.3, p = 1E−09)/stage (cor = 0.32, p = 1E−10). We selected 128 key genes (Fig. 5c) in the turquoise module according to the standard. To explore the potential function of these key genes, pathway and process enrichment analysis of these key genes were performed, as shown in Fig. 5d. The three most highly significantly enriched terms were extracellular matrix organization, collagen fibril organization, and ECM proteoglycans, all related to the tumor microenvironment (TME).

### Immune cells can be combined with risk scores for prognostic analysis

**Fig. 6:**
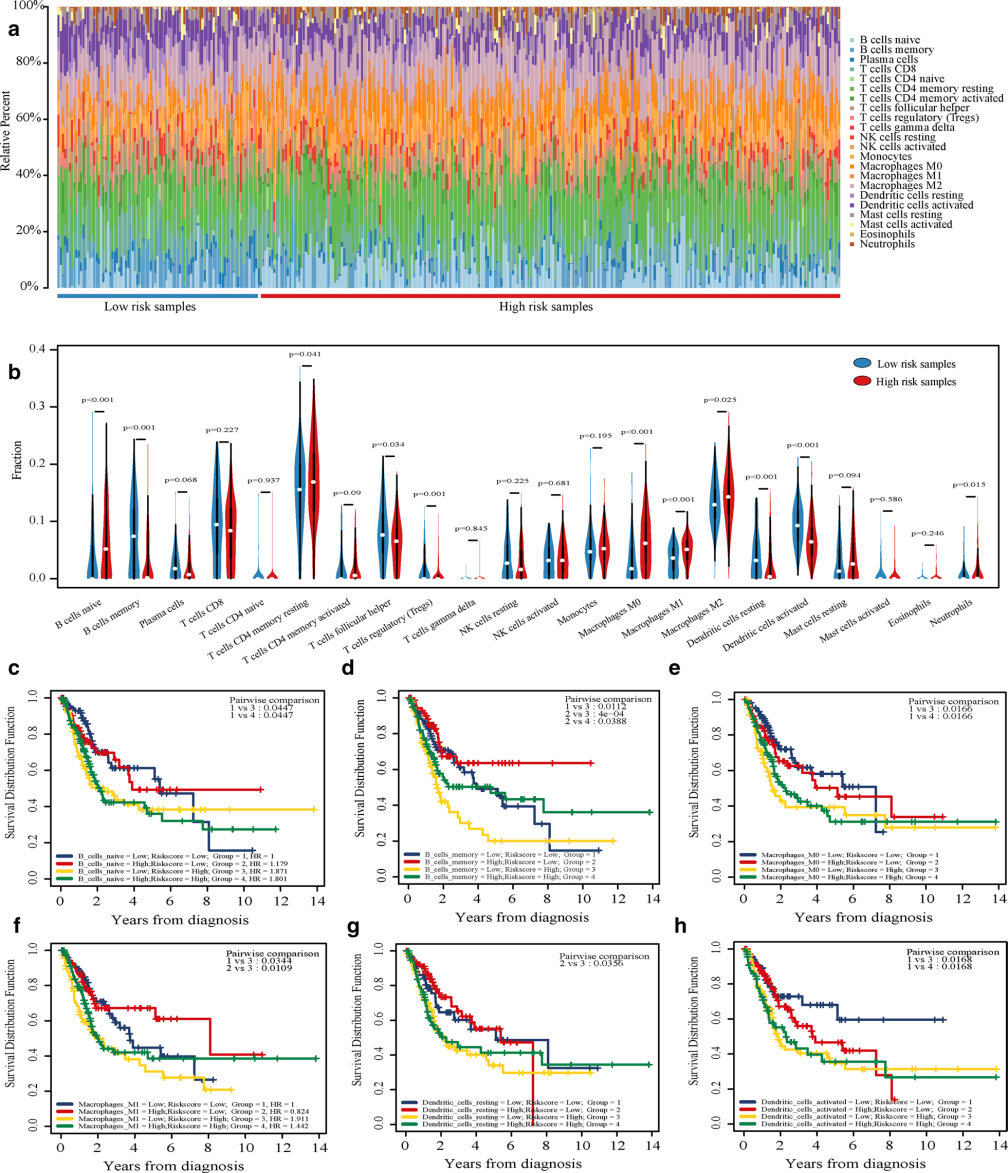
Combined analysis of risk score and immune infiltrating cells. **a** The bar chart of the infiltration of 22 kinds of immune cells in the samples of high and low-risk groups. **b** The difference of 22 kinds of immune cells between the low-risk group and the high-risk group was analyzed and shown by violin chart. p < 0.05 indicated that it was statistically significant. **c**–**h** Combined Kaplan–Meier analysis of 6 kinds of immune cells and risk scores

We used CIBERSORT to calculate the infiltration ratio of 22 immune cells in TCGA-BLCA samples and used a bar chart to show the infiltration of high and low-risk groups (Fig. 6a). Then, the Wilcoxon test was used to compare the difference between high and low-risk groups. The results showed that B cells naive, Macrophages M0, and Macrophages M1 showed high infiltration in the high-risk group; B cells memory, Dendritic cells resting, and Dendritic cells activated showed high infiltration in the low-risk group (p < 0.001, Fig. 6b). Furthermore, we took the risk score and the infiltration degree of these six kinds of immune cells for joint prognostic analysis. The samples were divided into four clusters for Kaplan–Meier analysis according to the median value of the risk score and immune cell infiltration degree. The results showed that these groups could also be used for prognostic analysis (p < 0.05, Fig. 6c–h). Among them, the prognostic ability of B cells memory is the best. When the degree of B cells memory infiltration is low, and the risk score is high, the prognosis of this cluster is significantly worse than that of other clusters. We used ESTIMATE to calculate the TCGA-BLCA cohort’s immune score and then combined with the risk score for Kaplan–Meier analysis. The results showed that the cluster with low immune-score and high risk-score had the worst prognosis (Additional file 8: Figure S5).

## Discussion

Bladder cancer is a heterogeneous disease with a high incidence and recurrence rate, but there is no robust predictive tool to guide clinical treatment [5]. Some recent studies have also constructed a new model for bladder cancer, such as DNA methylation-driven genes related model [23] and immune genes related model [10]. These models prefer to take a kind of gene set to build the model, rather than the whole genome into the screening. In this study, prognostic genes were screened from four cohorts with the whole transcriptome, and the common prognostic genes were selected to construct the model. The model successfully predicted the overall survival of five cohorts about 1000 bladder cancer patients, and it is the research with the largest cohort size in the same type of research.

A variety of regional source cohorts are used to jointly develop the model, which makes the model have higher credibility and broader applicability. In our study, all genes in all cohorts were then analyzed by univariate Cox proportional hazard analysis to screen common prognostic genes in four cohorts. After further screening, a prognostic model was constructed using the data from the TCGA-BLCA cohort. Instead of using the genes obtained by analysis of a single cohort to construct a prognostic model, the prognostic genes common to multiple cohorts were used to make the model more stable and reliable. The patients in the TCGA-BLCA cohort were from North America, GSE13507 was from Asia, and GSE32548 and GSE32894 were from Europe. It is concluded that this model has a wide range of applicability.

The main finding of this study is that the 11-gene model we developed has a robust prognostic ability and successfully predicted the prognosis of five cohorts. Kaplan–Meier analysis showed that the prognosis of the high-risk group was worse in all the four cohorts (p < 0.001). The 1-year AUC values of the TCGA-BLCA, GSE13507, GSE32548, and GSE32894 cohorts were 0.686, 0.800, 0.826 and 0.781 respectively, indicating that the risk score has the ability to predict prognosis. Univariate and multivariate Cox analysis of clinical factors and risk scores showed that the ability of risk scores to predict prognosis was better than age, gender, grade, and T stage. We also analyzed the relationship between risk score and different clinical status, and found increased risk score with the increase of bladder cancer T stage and grade (p < 0.001). There are also significant differences in risk scores between wild type and mutant types of different genes. We analyzed the GSE32548 mutation data and found lower risk score in the group of FGFR3 mutation. In contrast, in the presence of a TP53 mutation (or with MDM2 alteration), the risk score was higher. According to previous reports, mutations in FGFR3 [24] is associated with better prognosis, but TP53 mutation (or with MDM2 alteration) is associated with worse prognosis [25, 26]. These conclusions indirectly verify the prognostic ability of the risk model. Finally, the 11-gene model was successfully verified in independent cohort GSE48075. The model is verified by four internal cohorts and one external cohort, which shows that the model has the potential to be used in the clinic.

Eleven genes are potential prognostic markers and therapeutic targets for bladder cancer. These 11 genes have a stable prognostic ability in TCGA-BLCA, GSE13507, GSE32548, and GSE32894 cohorts. And Kaplan–Meier analysis showed that SERPINE2, RTKN, PRR11, MAPK12, ELOVL4, DSEL, and COMP was successfully verified in GSE48075. Besides, the methylation level of TNK1 and C16orf74 can also predict the prognosis of bladder cancer. Among them, only C16orf74 and RTKN were previously reported to be associated with bladder cancer, while other genes were not reported and are worthy of future research for bladder cancer. SERPINE2 can enhance the tumor-promoting effect of ERK signal transduction in intestinal epithelial cells and is a potential therapeutic target for colorectal cancer [27]. This gene can also drive distant metastasis of breast cancer [28]. PRR11 is overexpressed in ovarian cancer [29], and has the potential to be used as a molecular marker. FABP6 is overexpressed in colon cancer and may play an important role in early carcinogenesis [30]. Decreased expression of C16orf74 is closely related to the progression of non-muscular invasive bladder cancer [31], and it may also be a potential therapeutic target for pancreatic cancer [32]. Most of the studies of DSEL are studies of congenital diseases, such as diaphragmatic defect [33] and Ehlers-Danlos syndrome [34], but there have been very few studies related to cancer. DNM1 is a kinetin-related protein that plays an important role in mitochondrial division [35]. COMP is a cartilage biomarker [36], and COMP mutation can cause pseudochondrodysplasia [37]. TNK1 is a tumor suppressor that can down-regulate the activity of Ras [38]. Studies have shown that RTKN is highly expressed in bladder cancer [39], and some experiments have shown that some miRNA can inhibit tumor growth by targeting RTKN [40]. MAPK12, one of four types of p38 MAPK, is a potential therapeutic target for colon cancer [41]. ELOVL4 is a member of the fatty acid elongation enzyme ELOVL family and is highly methylated in cancers such as hepatocellular carcinoma [42]. These genes may be involved in the essential biological process of bladder cancer and have great research value.

The combination of risk score and B cell memory can be used to analyze the prognosis of patients with bladder cancer. In the present study, the key genes positively related to the risk score were identified by WGCNA. The enrichment analysis of these genes showed that these genes were related to TME, s indicating that the patients’ risk core was closely related to TME. To further explore the relationship between risk score and TME, we calculated the infiltration degree of 22 kinds of immune cells in the sample. We found that there were differences in the infiltration degree of many immune cells between high and low risk. B cells memory stands out in the evaluation of combined immune cell and risk prognostic analysis, and the prognosis of patients is the worst in the case of low infiltration and high risk. Tumor-infiltrating lymphocytes have been reported as a useful prognostic factor for patients with bladder cancer [43], and B cells are a significant component of infiltration in these cells. B cell is a prognostic factor in many cancers (such as high grade serous ovarian cancer) [44]. CD20 B cells have also been reported to be associated with longer survival in bladder cancer [45]. Bladder cancer has a certain response to immunotherapy, but there is a lack of unique immune prognostic biomarker to guide treatment [46]. We combine B cell memory and risk score for prognostic analysis, which has prognostic ability and potential for immunotherapy.

Although this 11-gene risk prognostic model can predict the prognosis of bladder cancer, there are still several limitations to our conclusions. We used pre-existing data from available databases and did not verify the model with additional data. We did not find the general cut-off value of the model, so when the model is applied to the clinic, it needs to conduct a large local sample study to find the best cut-off for the cohort.

## Conclusions

The 11-genes model is a robust biomarker for the prognosis of bladder cancer, which can be used to predict patients’ survival outcomes. Future studies need to verify the accuracy of its prediction and clinical application in the individualized treatment of bladder cancer.

## Supplementary information

**Additional file 1: Table S1.** The basic information and coef values for the selected genes.

**Additional file 2: Table S2.** The average expression value of the selected genes in four cohorts.

**Additional file 3: Table S3.** Eleven Genes were analyzed by univariate Cox regression in the four cohorts.

**Additional file 4: Figure S1.** Methylation exploration of 11 genes. The co-expression between DNA methylation and gene expression of COMP (**a**), DNM1 (**b**), FABP6 (**c**), SERPINE2 (**d**), RTKN (**e**), MAPK12 (**f**), ELOVL4 (**g**), TNK1 (**h**), PRR11 (**i**), DSEL (**j**), C16orf74 (**k**). The Kaplan–Meier analysis of TKN1 (**l**) and C16orf74 (**m**) with the DNA methylation level.

**Additional file 5: Figure S2.** Risk score analysis of four cohorts. **a** TCGA-BLCA risk score analysis from top to bottom: patient’s risk distribution, gene expression profile and survival status map. **b** GSE13507. **c** GSE32548. **d** GSE32894.

**Additional file 6: Figure S3.** Differences of risk scores among different clinical conditions. **a** The differences of risk scores with different pathological grades for the four cohorts. **b** The differences of risk scores with different T stages for the four cohorts. **c** The difference of risk scores between wild type and mutant type in the GS32548 cohort. P < 0.05 is considered statistically significant.

**Additional file 7: Figure S4.** Kaplan–Meier analysis of the 11 genes in GSE48075. Kaplan–Meier analysis of TNK1(**a**), SERPINE2(**b**), RTKN(**c**), PRR11(**d**), MAPK12(**e**), FABP6(**f**), ELOVL4(**g**), DSEL(**h**), DNM1(**i**), COMP(**j**), and C16orf74(**k**). P-value shows green when p < 0.05. Hazard Radio (HR) shows red when HR > 1 and shows blue when HR < 1.

**Additional file 8: Figure S5.** Kaplan–Meier analysis of risk score and immune score. According to the median risk score and immune score, the patients were divided into 4 clusters for Kaplan–Meier analysis. P < 0.05 indicated that it was statistically significant.

## Data Availability

The TCGA-BLCA dataset used in this study could be obtained from TCGA database (https://cancergenome.nih.gov/). Four GEO datasets (GSE31507, GSE32548, GSE32894, and GSE40875) used in this study could be obtained from GEO database (https://www.ncbi.nlm.nih.gov/geo/).

## Abbreviations

TCGA: The Cancer Genome Atlas
GEO: Gene Expression Omnibus
BLCA: Bladder urothelial carcinoma
ROC: Receiver operating characteristic
AUC: Area under the curve
HR: Hazard ratio
Lasso: Least absolute shrinkage and selection operator
coef: Regression coefficient
TME: Tumor microenvironment
