# Supplementary material for: A robust 11-genes prognostic model can predict overall survival in bladder cancer patients based on five cohorts
Source: Cancer Cell Int. 2020 Aug 20;20:402. doi: 10.1186/s12935-020-01491-6 (PMC7441568; doi:10.1186/s12935-020-01491-6)
Supplement: Supplementary file 1 — Additional file 1: Table S1. The basic information and coef values for the selected genes. [file 12935_2020_1491_MOESM1_ESM.docx]

Table S1. The basic information and coef values for the selected genes.

| Gene stable ID | Gene name | Gene type | Chromosome | Gene start (bp) | Gene end (bp) | Coef |
| --- | --- | --- | --- | --- | --- | --- |
| ENSG00000135919 | *SERPINE2* | Protein Coding | 2 | 223,975,045 | 224,039,319 | 2.00E-02 |
| ENSG00000068489 | *PRR11* | Protein Coding | 17 | 59,155,499 | 59,206,709 | 1.30E-01 |
| ENSG00000170231 | *FABP6* | Protein Coding | 5 | 160,187,367 | 160,238,735 | 3.18E-04 |
| ENSG00000154102 | *C16orf74* | Protein Coding | 16 | 85,690,084 | 85,751,129 | 5.64E-02 |
| ENSG00000171451 | *DSEL* | Protein Coding | 18 | 67,506,582 | 67,516,730 | 1.07E-01 |
| ENSG00000106976 | *DNM1* | Protein Coding | 9 | 128,191,655 | 128,255,249 | 1.42E-02 |
| ENSG00000105664 | *COMP* | Protein Coding | 19 | 18,782,773 | 18,791,305 | 2.23E-02 |
| ENSG00000174292 | *TNK1* | Protein Coding | 17 | 7,380,534 | 7,389,774 | -9.72E-02 |
| ENSG00000118402 | *ELOVL4* | Protein Coding | 6 | 79,914,812 | 79,947,598 | 1.52E-03 |
| ENSG00000114993 | *RTKN* | Protein Coding | 2 | 74,425,836 | 74,442,422 | 1.26E-01 |
| ENSG00000188130 | *MAPK12* | Protein Coding | 22 | 50,245,450 | 50,261,759 | 3.04E-02 |
