# Supplementary material for: A robust 11-genes prognostic model can predict overall survival in bladder cancer patients based on five cohorts
Source: Cancer Cell Int. 2020 Aug 20;20:402. doi: 10.1186/s12935-020-01491-6 (PMC7441568; doi:10.1186/s12935-020-01491-6)
Supplement: Supplementary file 2 — Additional file 2: Table S2. The average expression value of the selected genes in four cohorts. [file 12935_2020_1491_MOESM2_ESM.docx]

Table S2. The average expression value of the selected genes in four cohorts.

| Gene name | TCGA-OV | GSE13507 | GSE32548 | GSE32894 |
| --- | --- | --- | --- | --- |
| *SERPINE2* | 12.84512305 | 9.482435625 | 7.831339346 | 8.078151695 |
| *PRR11* | 21.87308944 | 8.125093779 | 6.193634497 | 6.124023074 |
| *FABP6* | 32.40502035 | 9.758332627 | 7.424868045 | 8.094972924 |
| *C16orf74* | 37.86943378 | 9.11343447 | 6.34237581 | 6.692086861 |
| *DSEL* | 1.077323919 | 7.534021682 | 5.358546857 | 5.581577969 |
| *DNM1* | 6.330609177 | 7.742449154 | 5.320370704 | 5.376012447 |
| *COMP* | 73.82551608 | 7.945122019 | 5.616406965 | 5.736705441 |
| *TNK1* | 17.85912244 | 7.972737275 | 5.375372447 | 5.54842872 |
| *ELOVL4* | 4.470982647 | 7.767103924 | 6.100555502 | 6.184319131 |
| *RTKN* | 26.84925238 | 9.378970099 | 6.517711673 | 6.594588691 |
| *MAPK12* | 3.321595435 | 7.429358985 | 5.362122744 | 5.591792291 |
