# Supplementary material for: A robust 11-genes prognostic model can predict overall survival in bladder cancer patients based on five cohorts
Source: Cancer Cell Int. 2020 Aug 20;20:402. doi: 10.1186/s12935-020-01491-6 (PMC7441568; doi:10.1186/s12935-020-01491-6)
Supplement: Supplementary file 3 — Additional file 3: Table S3. Eleven Genes were analyzed by univariate Cox regression in the four cohorts. [file 12935_2020_1491_MOESM3_ESM.docx]

Table S3. Eleven Genes were analyzed by univariate Cox regression in the four cohorts.

| Genes | TCGA_BLCA | | GSE13507 | | GSE32548 | | GSE32894 | |
| --- | --- | --- | --- | --- | --- | --- | --- | --- |
|  | HR (95% CI) | P | HR (95% CI) | P | HR (95% CI) | P | HR (95% CI) | P |
| *SERPINE2* | 1.13(1.05-1.22) | 1.81E-03 | 1.32(1.12-1.54) | 6.98E-04 | 1.53(1.22-1.92) | 2.57E-04 | 1.64(1.3-2.06) | 2.48E-05 |
| *PRR11* | 1.23(1.05-1.44) | 8.71E-03 | 1.45(1.09-1.94) | 1.16E-02 | 2.08(1.13-3.81) | 1.83E-02 | 3.62(1.97-6.63) | 3.33E-05 |
| *FABP6* | 0.54(0.41-0.72) | 2.18E-05 | 0.84(0.72-0.99) | 3.92E-02 | 0.61(0.45-0.83) | 1.66E-03 | 0.54(0.41-0.72) | 2.18E-05 |
| *C16orf74* | 0.35(0.2-0.63) | 4.02E-04 | 0.7(0.54-0.92) | 8.75E-03 | 0.54(0.31-0.96) | 3.54E-02 | 0.35(0.2-0.63) | 4.02E-04 |
| *DSEL* | 1.23(1.11-1.37) | 1.27E-04 | 1.59(1.01-2.51) | 4.35E-02 | 5.9(1.83-19.03) | 2.98E-03 | 4.84(1.69-13.87) | 3.35E-03 |
| *DNM1* | 1.17(1.05-1.3) | 3.33E-03 | 1.74(1.25-2.42) | 1.06E-03 | 4.54(2-10.29) | 2.94E-04 | 6.11(3.06-12.2) | 2.87E-07 |
| *COMP* | 1.07(1.02-1.12) | 9.72E-03 | 1.41(1.14-1.74) | 1.47E-03 | 1.49(1.14-1.96) | 3.96E-03 | 1.72(1.32-2.23) | 5.29E-05 |
| *TNK1* | 0.03(0-0.24) | 1.17E-03 | 0.49(0.27-0.89) | 1.81E-02 | 0.06(0-0.83) | 3.56E-02 | 0.03(0-0.24) | 1.17E-03 |
| *ELOVL4* | 1.14(1.04-1.23) | 2.94E-03 | 1.63(1.27-2.09) | 1.38E-04 | 1.81(1.21-2.71) | 3.79E-03 | 2.52(1.62-3.91) | 4.05E-05 |
| *RTKN* | 1.23(1.07-1.42) | 3.74E-03 | 1.36(1.04-1.78) | 2.24E-02 | 1.92(1.11-3.32) | 1.97E-02 | 2.58(1.46-4.55) | 1.04E-03 |
| *MAPK12* | 1.17(1.04-1.32) | 9.95E-03 | 1.89(1.31-2.72) | 6.01E-04 | 2.41(1.07-5.47) | 3.46E-02 | 3.39(1.67-6.89) | 7.53E-04 |

HR, hazard ratio; CI, confidence interval.
