# Supplementary figures and images for: A robust 11-genes prognostic model can predict overall survival in bladder cancer patients based on five cohorts
Source: Cancer Cell Int. 2020 Aug 20;20:402. doi: 10.1186/s12935-020-01491-6 (PMC7441568; doi:10.1186/s12935-020-01491-6)

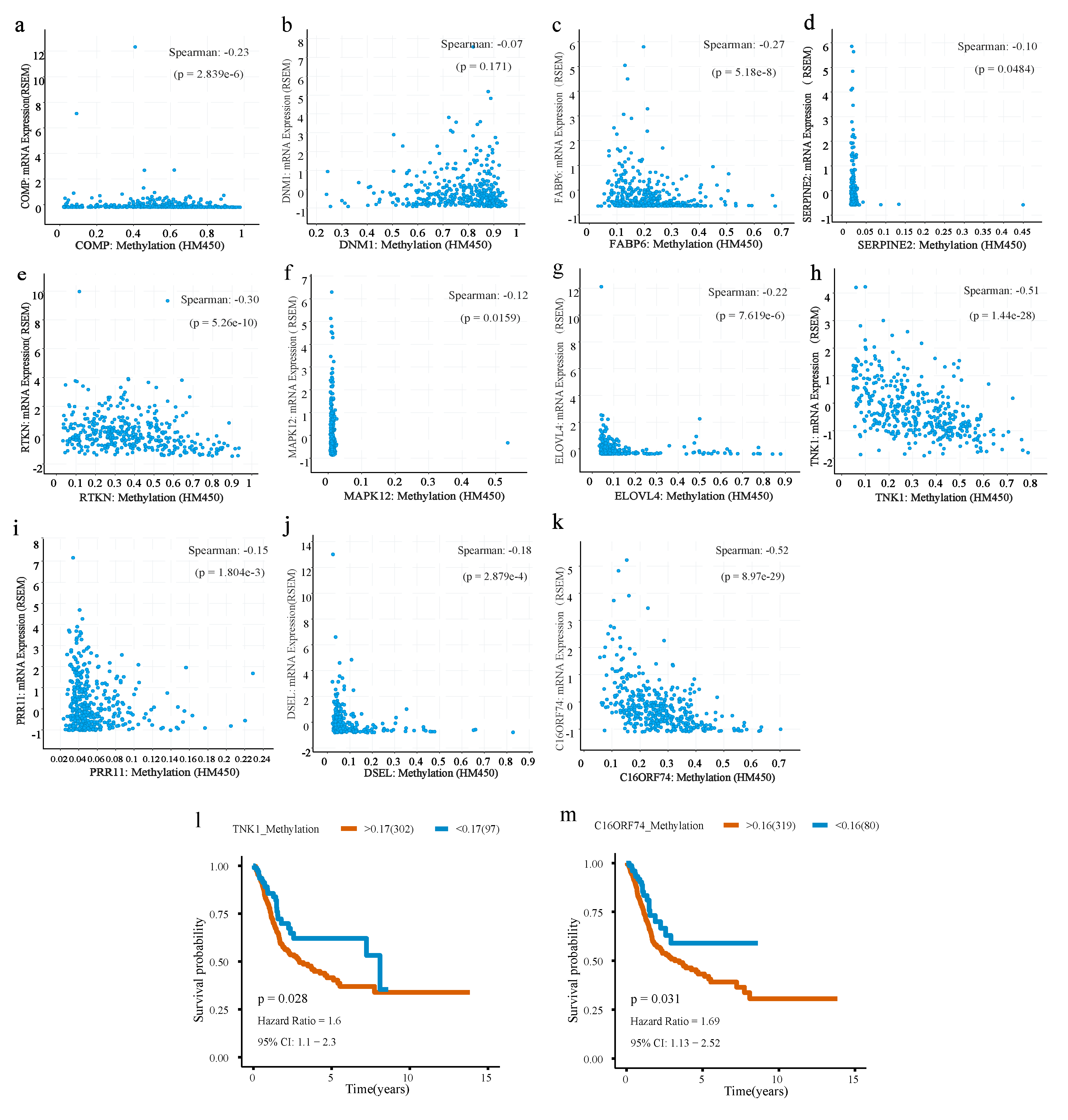

Supplement: Supplementary file 4 — Additional file 4: Figure S1. Methylation exploration of 11 genes. The co-expression between DNA methylation and gene expression of COMP (a), DNM1 (b), FABP6 (c), SERPINE2 (d), RTKN (e), MAPK12 (f), ELOVL4 (g), TNK1 (h), PRR11 (i), DSEL (j), C16orf74 (k). The Kaplan–Meier analysis of TKN1 (l) and C16orf74 (m) with the DNA methylation level. [file 12935_2020_1491_MOESM4_ESM.tif]

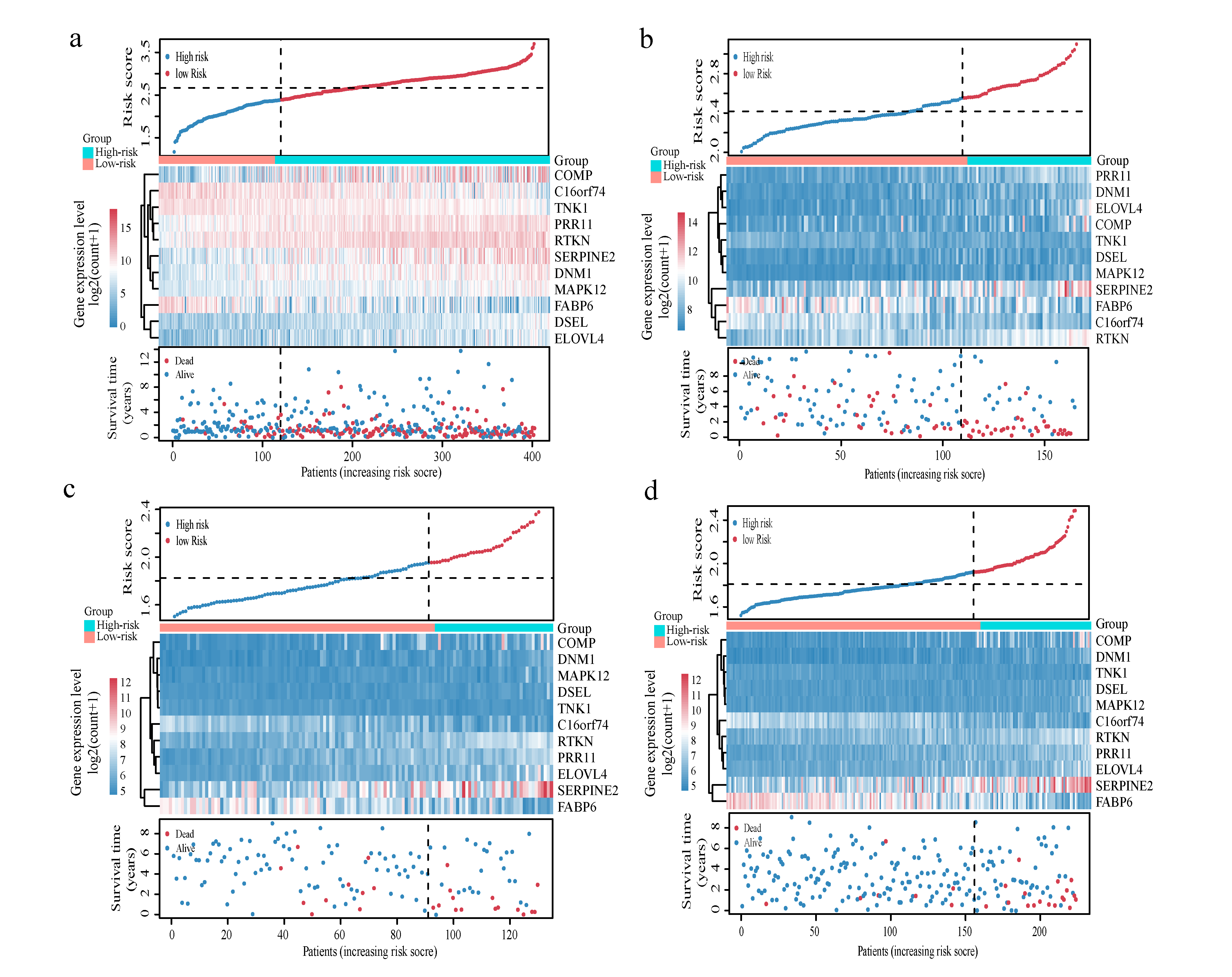

Supplement: Supplementary file 5 — Additional file 5: Figure S2. Risk score analysis of four cohorts. a TCGA-BLCA risk score analysis from top to bottom: patient’s risk distribution, gene expression profile and survival status map. b GSE13507. c GSE32548. d GSE32894. [file 12935_2020_1491_MOESM5_ESM.tif]

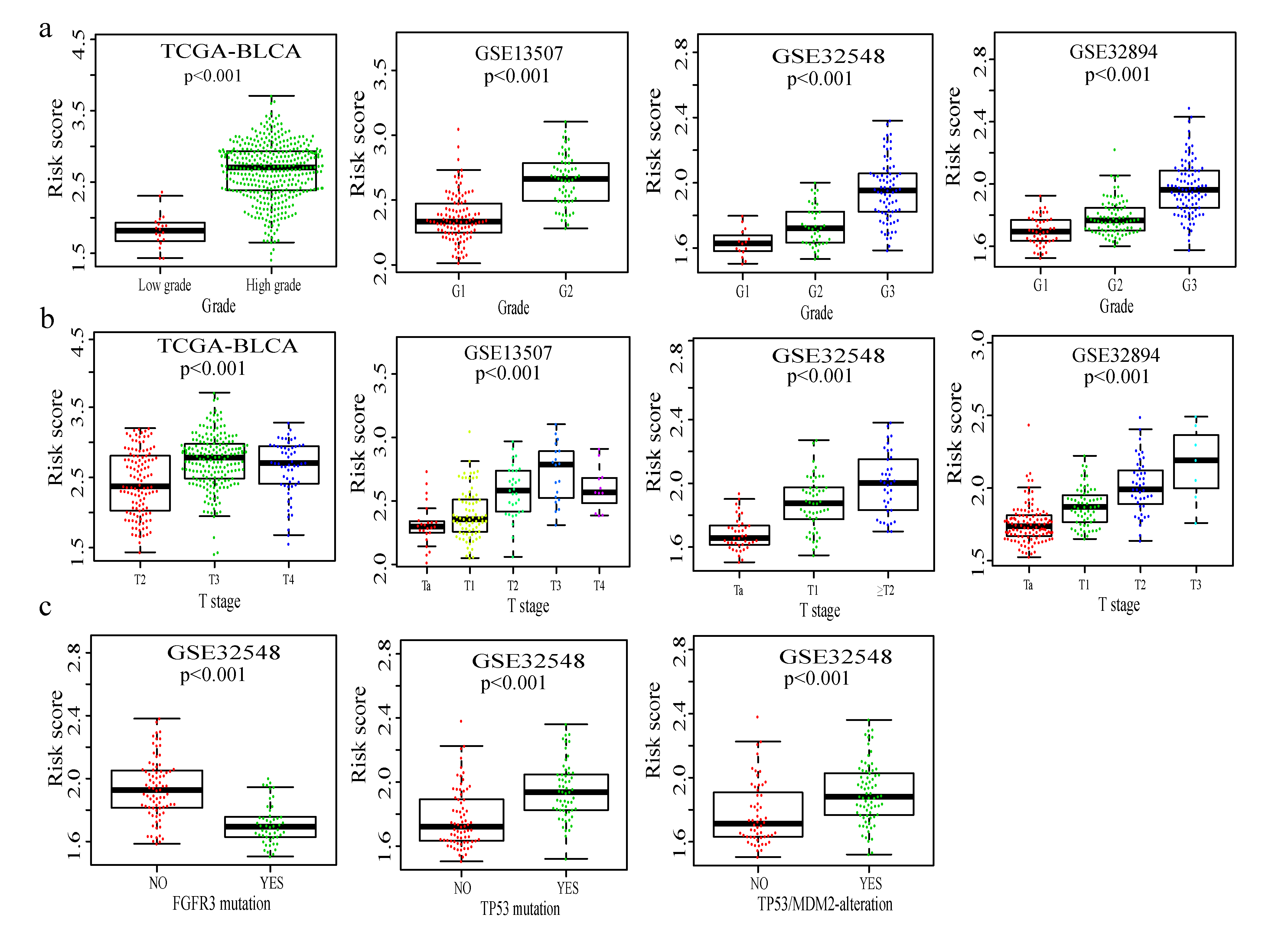

Supplement: Supplementary file 6 — Additional file 6: Figure S3. Differences of risk scores among different clinical conditions. a The differences of risk scores with different pathological grades for the four cohorts. b The differences of risk scores with different T stages for the four cohorts. c The difference of risk scores between wild type and mutant type in the GS32548 cohort. P < 0.05 is considered statistically significant. [file 12935_2020_1491_MOESM6_ESM.tif]

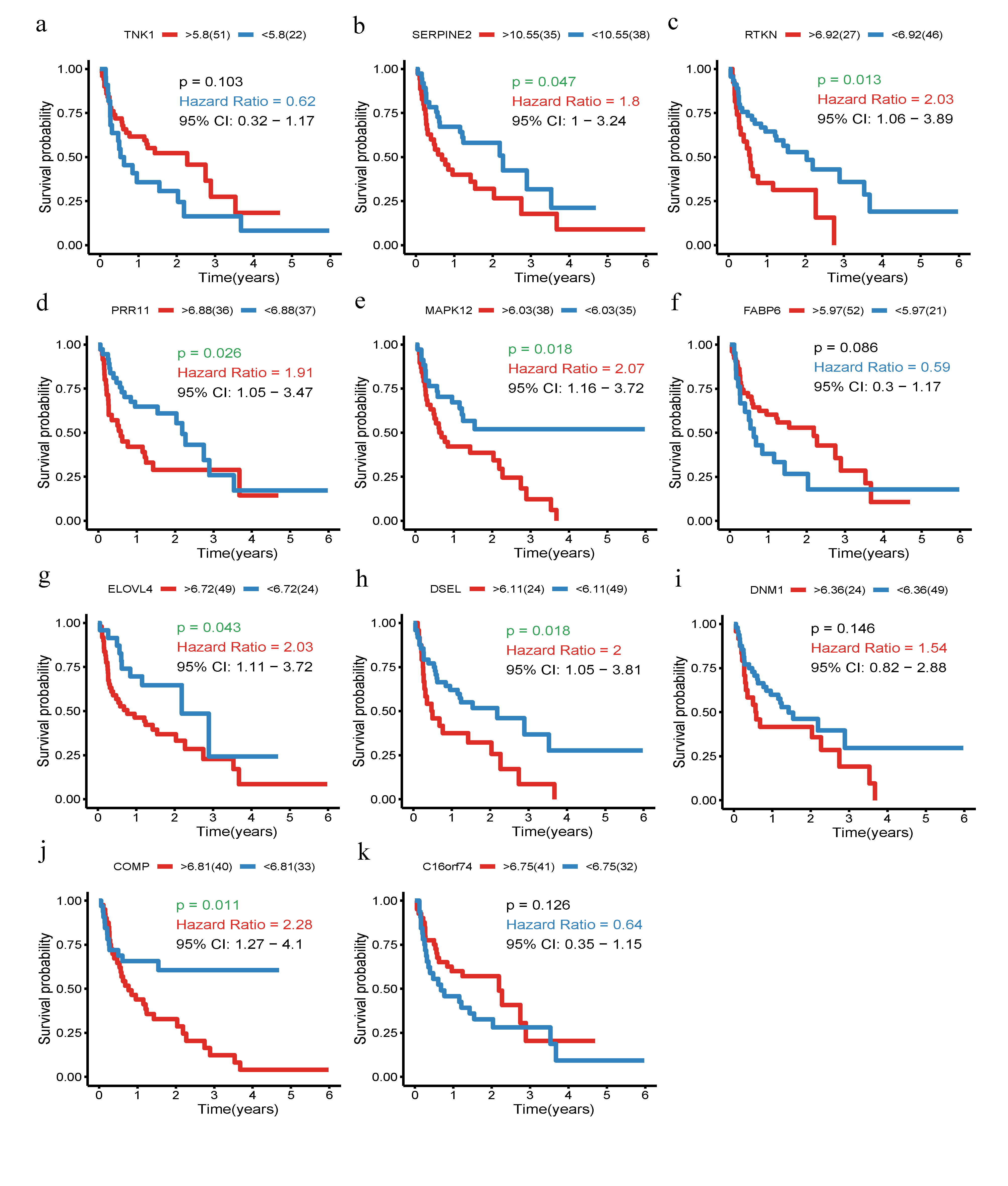

Supplement: Supplementary file 7 — Additional file 7: Figure S4. Kaplan–Meier analysis of the 11 genes in GSE48075. Kaplan–Meier analysis of TNK1(a), SERPINE2(b), RTKN(c), PRR11(d), MAPK12(e), FABP6(f), ELOVL4(g), DSEL(h), DNM1(i), COMP(j), and C16orf74(k). P-value shows green when p < 0.05. Hazard Radio (HR) shows red when HR > 1 and shows blue when HR < 1. [file 12935_2020_1491_MOESM7_ESM.tif]

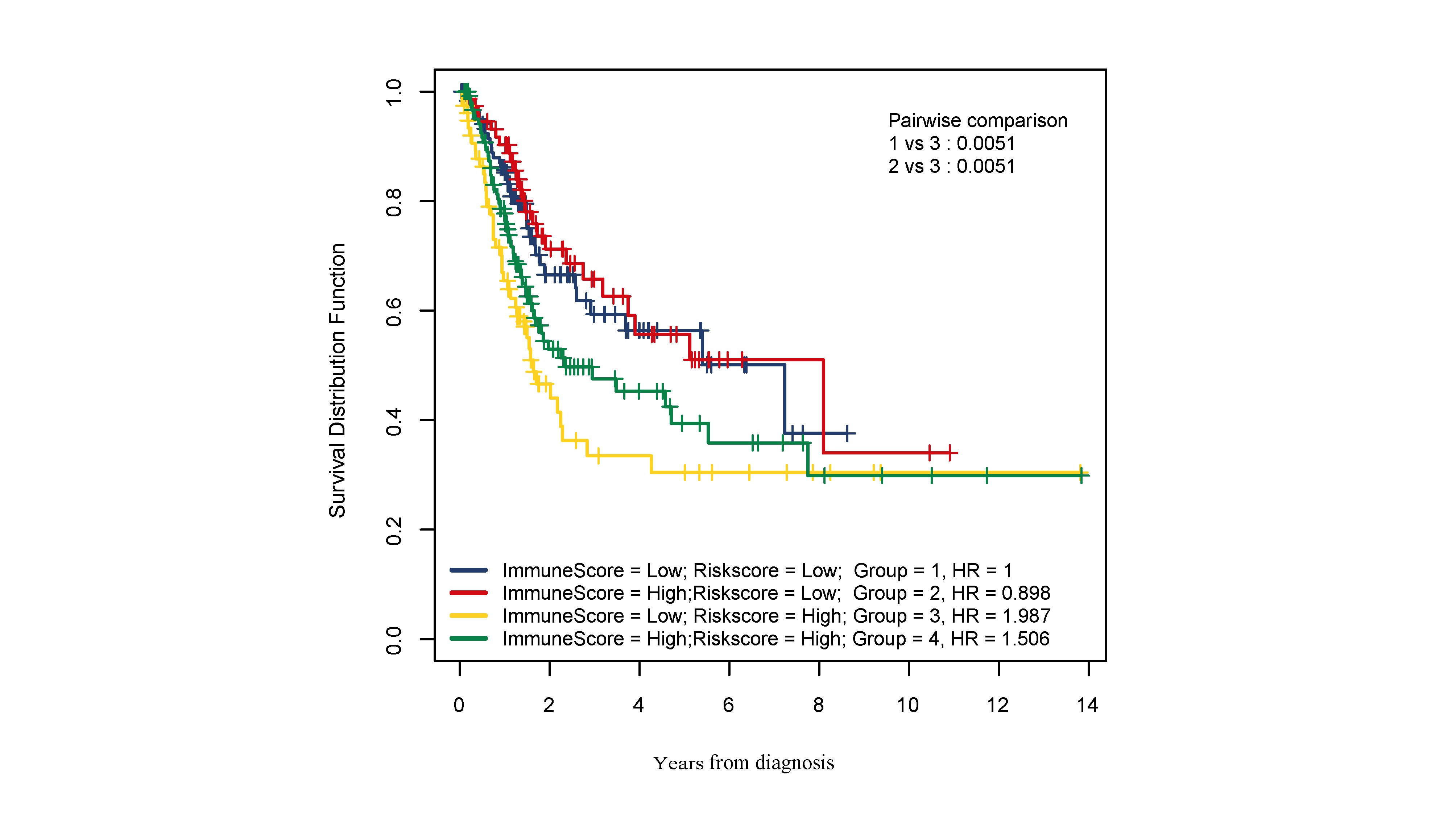

Supplement: Supplementary file 8 — Additional file 8: Figure S5. Kaplan–Meier analysis of risk score and immune score. According to the median risk score and immune score, the patients were divided into 4 clusters for Kaplan–Meier analysis. P < 0.05 indicated that it was statistically significant. [file 12935_2020_1491_MOESM8_ESM.tif]
